# An Integrated Pipeline of Open Source Software Adapted for Multi-CPU Architectures: Use in the Large-Scale Identification of Single Nucleotide Polymorphisms

**DOI:** 10.1155/2007/35604

**Published:** 2007-12-02

**Authors:** B. Jayashree, Manindra S. Hanspal, Rajgopal Srinivasan, R. Vigneshwaran, Rajeev K. Varshney, N. Spurthi, K. Eshwar, N. Ramesh, S. Chandra, David A. Hoisington

**Affiliations:** ^1^Bioinformatics Unit, GT-Biotechnology, International Corps Research Institute for the Semi-Arid Tropics, Patancheru 502324, India; ^2^Advanced Technology Centre, Tata Consultancy Services, Madhapur, Hyderabad 500081, India; ^3^Applied Genomics Laboratory, GT-Biotechnology, International Corps Research Institute for the Semi-Arid Tropics, Patancheru 502324, India; ^4^Novell IDE, Bangalore 560068, India

## Abstract

The large amounts of EST sequence data available from a single species of an organism as well as for several species within a genus provide an easy source of identification of intra- and interspecies single nucleotide polymorphisms (SNPs). In the case of model organisms, the data available are numerous, given the degree of redundancy in the deposited EST data. There are several available bioinformatics tools that can be used to mine this data; however, using them requires a certain level of expertise: the tools have to be used sequentially with accompanying format conversion and steps like clustering and assembly of sequences become time-intensive jobs even for moderately sized datasets. We report here a pipeline of open source software extended to run on multiple CPU architectures that can be used to mine large EST datasets for SNPs and identify restriction sites for assaying the SNPs so that cost-effective CAPS assays can be developed for SNP genotyping in genetics and breeding applications. At the International Crops Research Institute for the Semi-Arid Tropics (ICRISAT), the pipeline has been implemented to run on a Paracel high-performance system consisting of four dual AMD Opteron processors running Linux with MPICH. The pipeline can be accessed through user-friendly web interfaces at http://hpc.icrisat.cgiar.org/PBSWeb and is available on request for academic use. We have validated the developed pipeline by mining chickpea ESTs for interspecies SNPs, development of CAPS assays for SNP genotyping, and confirmation of restriction digestion pattern at the sequence level.

## 1. INTRODUCTION

The bioinformatics analysis of large biological datasets demand solutions involving serialization of several steps into a pipeline as well as parallelization of certain steps within the pipeline. One such example is the analysis of nucleotide datasets such as expressed sequence tags (ESTs) for identification of sequence variations or single nucleotide polymorphisms (SNPs), which can be used as molecular markers in genetics and breeding applications. A number of sequential steps are involved, each of which need to be performed with a different bioinformatics tool. The tools can be implemented within a pipeline, with code to take care of intermediate tasks like data format conversions, parsing of output files, and summarizing output files. For large datasets, these pipelines may need to be constructed so as to permit parallel processing of some or all of the steps within a pipeline, thereby reducing the time taken for job completion as well as to handle common memory problems that occur when using the pipeline on a single processor. Mining of SNPs in large datasets of ESTs is one such problem that may require parallel processing of some steps of the pipeline.

Single nucleotide polymorphisms (SNPs) are highly stable genetic markers that can be used in the study of complex genetic traits and genome evolution [1]. Mining for SNPs from EST sequences makes for cost-effective identification of polymorphisms especially for those organisms where there is little genome sequence data available. One of the main advantages of using ESTs is that markers closely linked with or directly in the coding regions of genes can be identified; generating maps increasingly populated with gene-associated markers [2]. SNPs in ESTs can also identify sequence variants that lead to amino acid substitutions and perhaps lead to functional differences that could be associated with phenotypic effects. Projects driven by a need to reduce sequencing costs when it comes to generating high-density linkage maps, or the need to study the interspecies polymorphisms for evolutionary relationships between species would mine SNPs electronically from existing data. This is feasible only where sequence data is available, such as in the model organisms, and offers the possibility of extending to related but less-studied species. Several serial software pipelines have been reported in the literature and have been shown to be useful for datasets from specific model organisms.

Identifying SNPs from EST data involves the steps of sequence clustering and assembly followed by detection. Several software pipelines have been published, many of which are available open source for use by the community. Most pipelines have been designed specifically for resequencing projects; using data quality scores like those derived from Phred and thus requiring trace data. One popular tool is Polybayes [3] that implements a Bayesian statistical model for the rigorous treatment of sequence variation within a multiple alignment taking into account the quality values of the sequences and a priori expected rate of polymorphic sites in the region with custom scripts for sequence clustering. The tool SSAHA SNP requires that raw whole genome sequence be available to be searched against mRNA and EST sequence databases, and functions by organizing the database to be searched into a hash table data structure [4]. The PERL package POSA [5] implements the tools Polyphred and Polybayes in a pipeline while another pipeline that uses Phrap, CAT, and Polybayes [6] was tested with the maize EST dataset optimizing the Polybayes algorithm to work without sequence data quality values. The more recent SNP PHAGE (SNP discovery pipeline with additional features for haplotype analysis and genbank submissions [7]) is a modification of the PERL package POSA. The novoSNP [8] program allows automated, fast identification of variation from trace files using a reference sequence; and the miraEST assembler detects SNPs during assembly but needs trace files [9]. These are some of the pipelines reported in the literature and many of them are dependent on the availability of sequence trace data. Software approaches to mine for SNPs from EST sequence alignments without the requirement for trace files include AutoSNP [10] and the SNiPpER algorithms that were used for identification of SNPs from large EST collections in barley [11]. While these are serial software; algorithms that help execute the time-intensive processes of clustering and assembly faster have become available, such as the PaCE algorithm for the clustering of ESTs [12] and PCAP for assembly [13].

We report here another pipeline of public domain tools but with a difference. It is a web-based application wherein all the tools in the pipeline have been implemented within a parallel framework and can work on beowulf clusters and SMP's. The pipeline outputs and their use would vary depending upon the combination of pipeline component software used. Clustering and assembly of very large EST datasets can be accomplished, which could be used in gene expression studies, differential gene expression studies, and in the identification of unigenes. The pipeline can be used to identify SNPs and also convert identified SNPs into cleaved amplified polymorphic sequences (CAPS) markers. The pipeline can be accessed either through user-friendly interfaces or the command line option. The validation of the SNPs predicted through this pipeline has been shown using a set of chickpea ESTs; and the optimized CAPS assays have been confirmed by sequencing the restriction digested fragments.

## 2. METHODS

### 2.1. Establishment of pipeline

The pipeline uses the open source tools MegaBlast for clustering [14], PCAP for assembly [13], Poybayes for SNP identification [3], and SNP2CAPS [15]. MegaBLAST, Polybayes, and SNP2CAPS have been implemented within a parallel framework with the MPICH implementation of message passing interface (MPI), because MPI is one of the most popular standards for writing parallel programs, efficiently manages message buffers, and has more than one freely available quality implementation. The MPICH wrappers were written in the python programming language. The modified version of MegaBLAST carries out clustering by performing an all versus all pair-wise comparison wherein a large database is split into slices and each slice is compared against the whole database in parallel. Results are merged and sorted using decreasing pair-wise alignment as the score. In the pipeline, the dataset is split into many fragments and given to processors as and when they finish comparing the previous fragment against the whole dataset. Since the time required to complete a sequence comparison depends on the similarity between them, the load is unevenly distributed if every processor is given a fixed set of fragments initially. To enable load balancing, fragments are assigned to processors as soon as they finish their previously assigned tasks. This assumes that the dataset is big enough to generate a large number of clusters. In addition, slave nodes can immediately inform the master upon completion of their assigned task. Thereafter, clustering is carried out using clustering utilities from TGICL: sclust, tclust, and nrcl [16]. The main idea behind clustering is to split the task of assembling the entire set of sequences into the assembling of several clusters of similar sequences. The assembly program PCAP is a parallel program. The time-consuming parts of the assembly, that is overlap detection and consensus generation, has been parallelized effectively in PCAP. However, PCAP carries out the assembly of each cluster using all the processors. The implementation of PCAP relies on the Portable Batch System (PBS) scheduler to spawn subjobs on other processors, which was considered unnecessary in the present application. Instead, clusters are simply submitted to a processor without distributing them; maximizing the use of the processor. Thus the MPI wrapper code helped achieve objectives while reducing dependency on a host of parallel programming tools. Polybayes is included in the pipeline for the detection of SNPs in the assembled EST sequences. This tool efficiently identifies sequence paralogs to avoid false predictions. Finally, the SNP2CAPS tool helps identify restriction sites for the SNPs.

The use of MPICH for writing wrappers, using the python programming language, makes the whole application portable. The pipeline includes PERL code for format conversion. The pipeline prerequisites are the modified version of MegaBlast, PCAP, Polybayes, and SNP2CAPS software programs. The web interfaces for the pipeline developed using OpenPBS http://www.openpbs.org use the Apache server, with a PostgreSQL database backend. The installation instructions are provided in the pipeline package.

### 2.2. Use of pipeline

#### 2.2.1. Validated dataset

While several datasets of different sizes were tested on the pipeline, the dataset that has been experimentally validated is a small dataset consisting of 1499 ESTs generated from twenty-six different *Cicer* species. This dataset was pipelined through the software for detection of interspecies SNPs. All the ESTs are available in the public domain.

#### 2.2.2. Plant material and DNA extraction

A total of 12 genotypes representing 8 *Cicer* species: *C. pungens* (ICC 17138), *C. bijugam* (ICC 17122), *C. microphyllum* (ICC 17248), *C. judaicum* (ICC 17148), *C. cuncetaum* (ICC 17162), *C. yamashitae* (ICC 17116), *C. pinnatifidum* (ICC 17152), *C. reticulatum* (ICC 17123 and PI 489777), and *C. arietinum* (ICC 8261, ICC 4958, and ICC 1882) were used for validation of the SNP pipeline. The DNA was extracted from two-week old seedlings using the protocol of Mace et al. [17].

#### 2.2.3. Polymerase chain reaction (PCR)

Amplifications were carried out in 20 *μ*L of reaction mixture containing 10 ng of genomic DNA, 1X PCR buffer, 1.5 mM MgCl_2_, 0.1 mM of dNTP mix, 0.2 mM of each primer, and 0.2 U of Taq DNA Polymerase (Bioline). Amplifications were performed in an Applied Biosystems thermal cycler using a touchdown amplification profile. The amplification cycles were: initial denaturation of 3 minutes at 95°C followed by 5 cycles of denaturation for 20 seconds at 94°C, touchdown from 60°C to 55°C with 1°C decrease in each cycle for 20 seconds followed by extension at 72°C for 30 seconds. The next 30 cycles were denaturation for 20 seconds at 94°C, annealing at 56°C for 20 seconds and extension at 72°C for 30 seconds followed by final extension of 20 minutes at 72°C and stored at 4°C until further use. The PCR products were run on 1.2% agarose to check for amplification.

#### 2.2.4. CAPS assays

10 *μ*L of the amplified products of final concentration of 100 ng were digested using 7.5 units of the restriction enzyme. The digested products were separated on 1.2% agarose gel electrophoresis.

#### 2.2.5. Sequencing of amplicons

The amplicons were purified using 1 unit of Exonuclease I and 1unit of shrimp alkaline phosphatase (SAP) per 5 *μ*L of PCR product. The Exo/SAP added PCR product was subjected to 37°C for 45 minutes and 80°C for 15 minutes in the thermal cycler. The Exo/SAP treated amplicons were mixed with 1 *μ*L of BigDye Terminator V3.1, 2 *μ*L of 5 X dilution buffer and 3.2 picomoles of primer (forward and reverse) and the volume was made to 10 *μ*L. The sequencing PCR profile was as follows: initial denaturation of 96°C for 30 seconds, followed by 60 cycles of 96°C for 10 seconds, 50°C for 5 seconds, and 60°C for 4 minutes. All reactions were stored at 4°C until further use.

The products were precipitated using 2.5 *μ*L of 125 mM EDTA and 25 *μ*L of absolute ethanol and incubated for 15 minutes at room temperature. The plate was spun at 4000 rpm for 30 minutes at 4°C and inverting the plate on tissue poured off the Ethanol/EDTA mix. To each well, 60 *μ*L of 70% ethanol was added and again spun at 4000 rpm for 20 minutes at 4°C. The ethanol was poured off as earlier. The plate was air-dried and 10 *μ*L of HiDi formamide added and the products denatured (94°C for 5 minutes, then immediately cooled to 4°C for 5 minutes) and sequenced using an ABI3700 automated sequencer.

The sequenced data along with the sequences of ESTs (that provided the SNPs initially) were aligned and analyzed using BioEdit.

## 3. Results

### 3.1. The software pipeline

**Figure 1:**
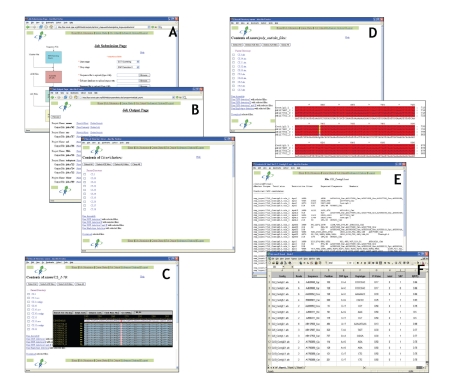
Web interfaces to the pipeline: (A) the job submission page; (B) retrieval of output files; (C) visualization of PCAP assemblies in Consed, (D) visualization of polybayes alignment files using Gendoc; (E) SNP2CAPS output; (F) example excel sheet returning number of haplotypes, INDELs, PIC, and *P* values from PCAP or polybayes output files.

The pipeline can be used to cluster and assemble very large EST datasets, return unigenes, identify SNP polymorphisms, and generate a table of SNPs, indels, number of reads, PIC value, haplotype, and so forth. Identified SNP polymorphisms can be also converted to CAPS markers if desired (see Figure 1). The advantage of implementing the pipeline to work on a cluster of machines is the ability to handle even very large input dataset size and assembly of large clusters (see Table 1) without running into time and memory problems as would happen on a desktop and achieving speedup with moderately sized datasets.

The steps of the pipeline with input and output at the end of each step are outlined.

- MegaBlast: input file consists of EST sequences in Fasta format. Files containing clusters of homologs are returned.
- Filtering: the output of the previous step can be filtered for clusters that have more than one genotype and can thus become the basis for identification of interspecies polymorphisms.
- PCAP: MegaBlast output can be submitted for assembly. Output consists of assembly results in .ace file format that can be visualized using freely available alignment editors (Gendoc/Consed). These files may be converted to the .aln format to serve as input to the SNP2CAPS program.
- Polybayes: output of PCAP serves as an input to this program. The program uses a python script to generate quality values if quality/trace data is not submitted. The output of this program in .aln file format can be visualized (Gendoc/Consed) or read by custom scripts that returns number of indels, haplotypes, polymorphism information content, and genetic variability (π) information from the alignment based on the method of SNiPpER [11]. The Polybayes tool may be skipped in the pipeline and, instead, the user can choose to identify sequence variations from the PCAP output.
- SNP2CAPS: the .aln files derived from PCAP or Polybayes can serve as an input to SNP2CAPS after converting data to a format acceptable by this tool. SNP2CAPS must be provided a rebase file to output plausible restriction sites and the associated fragment information from the alignment.

The software can be accessed through the GUI or through the command line. The web pages allow the user to submit jobs, view queue status, and retrieve output files from every step of the process (see Figure 1). The PCAP software has been implemented with a stringency level of 90–95% similarity per 100 bp, setting considered sufficient to prevent clustering of paralogous sequences. The user can identify clusters with sequences from two or more genotypes if interspecies SNPs are being sought, and choose to assemble clusters with only a single genotype if intraspecies sequence variants are sought. The SNPs identified may be viewed through the Consed interface or the alignment file may be opened in the Gendoc editor. Polybayes was used in the pipeline to overcome the variable sequence quality of the EST data, though this tool can be used only when the user has trace data or is aware of the low- and high-quality regions in his dataset. The script incorporated in the pipeline then generates base quality programmatically.

The script that identifies SNPs and reports haplotype from both PCAP and Polybayes output implements the following criteria: (i) potential SNPs are identified based on a redundancy criterion that every allele is represented by >1 sequence in a contig; (ii) all the sequences in a haplotype have the same nucleotide in every polymorphic site; (iii) the number of false positives predicted are reduced by the script that considers the alignment quality of the neighboring bases, searching a specified window size of 10 bases around each candidate single base-pair mismatch. The user's decision on the reliability of the identified polymorphism is further aided by the PIC value, number of haplotypes reported, and *P* values returned by the script. The PIC calculation is according to the method of Nei, and *P*-value calculations as given in the publication on SNiPpER [11].

### 3.2. Application-detection of SNPs from the public chickpea EST dataset

In order to demonstrate the utility of this SNP pipeline, a small dataset consisting of 1499 ESTs generated from twenty-six different *Cicer* species was pipelined through the software for detection of interspecies SNPs. All the ESTs are available in the public domain. The MegaBlast output resulted in 118 clusters, of which 11 clusters contained sequences from multiple species. These clusters could be assembled into 19 contigs, interspecific SNPs could be identified from the alignment in 15 contigs. Of 184 putative SNPs identified, the SNP2CAPS program predicted 73 CAPS markers. The SNP2CAPS output returned restriction sites to the SNPs and primers were designed using Primer3.

### 3.3. CAPS assays for SNP genotyping

To assay the identified SNPs in chickpea germplasm, the pipeline helped to identify the restriction enzyme sites for 73 of the 184 identified SNPs in the contigs. A total of eight primer pairs (CL3a, CL3c, CL3d-487, CL3e, CL4a, CL10, CL20, and CL99) were chosen for amplification based on predicted fragment length. The primer pairs were used to amplify each of the 12 genotypes. Since sometimes there was more than one restriction enzyme for assaying the SNP per primer, a total of 17 primer-restriction enzyme combinations (using the common restriction enzymes: *Xmn*I, *Nla*III, *Acc*I, *Aci*I, *Ban*I, *Hpa*II, *Xba*I, *Taq*I, *Eco*RV, *Rsa*I, *Sal*I, *Taq*I, *Bst*NI, and *Hae*III) were tested on the 12 chickpea genotypes. Out of 17 primer-enzyme combinations, restriction patterns were observed in the case of five primer-enzyme combinations. For example, *Aci*I and *Hae*III could restrict the amplicons obtained with CL3e primer. Similarly, the amplicons obtained with CL4a, CL20, CL99 showed restriction with *Eco*RV, *Bst*NI, and *Xba*I, respectively.

**Figure 2:**
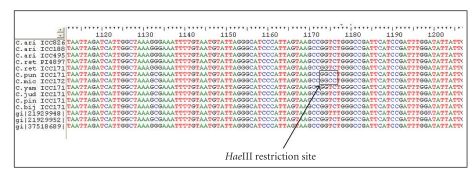
Multiple sequence alignment of the sequences obtained using CL3e primer in 11 *Cicer* genotypes.

In order to verify the SNPs at sequence level, the PCR amplicons for all 12 genotypes with CL3e primers were sequenced using the corresponding CL3e forward and reverse primers. Reasonably good-quality sequence data were obtained for 11 genotypes. These sequences, along with the sequences of three ESTs (AF522079, AF522081, and AY386897), which revealed the SNPs in the contig CL3e, were aligned and visualized for SNPs using BioEdit (see Figure 2). The sequence analysis showed the restriction site for *Hae*III (GGCC) in case of two genotypes (*C. microphyllum,* ICC 17248; and *C. pungens,* ICC 17138) out of 11 genotypes examined at position 1173 with respect to the original EST sequences. Indeed, these two genotypes showed restriction digestion in the CAPS assay. In addition, one more genotype (*C. yamashitae,* ICC 17116) showed restriction, however, at this particular position (i.e., 1173 bp), this genotype does not have an SNP. Nevertheless, we expect that the SNP should be present in the other direction to that which has been currently sequenced; we anticipate this after analyzing the sequence of three ESTs from which the SNP was derived.

## 4. DISCUSSION

Various tools are available that allow analysis of sequence datasets for polymorphism detection. A task like this, however, involves a number of sequential steps and substeps each of which needs to be performed with a different bioinformatic tool. Using these tools efficiently involves intermediate tasks like data format conversions, the need to write additional code for filters or to retrieve particular kinds of data, besides being time consuming. These are often problems when the dataset is large and not easily manageable. Putting large datasets through such pipelines may require processing of some tools in a parallel manner to allow faster run times. For this to be feasible either the individual subunits within software need to be parallelized if possible or MPI wrappers have to be implemented for the individual software components of the pipeline to permit them to function on clusters. The software pipeline reported in this paper does exactly this by allowing the user to run some or all steps of the process in parallel. The modified MegaBlast component of the pipeline speeds up the clustering process owing to a greedy algorithm and batch processing, in this pipeline it is implemented with MPI. The program PCAP can process several millions of reads and use multiple processors for sequence assembly. In this pipeline, this program has been implemented with an improved MPI wrapper. While the results in Table 1 are derived from a Paracel cluster of four 64-bit AMD Opteron processors available at ICRISAT, the pipeline can be executed both on beowulf clusters and SMPs.

Most software avoid the problem of large clusters by using a maximum cluster size of 20–50 for SNP discovery. The pipeline is not limited by cluster size as can be seen from Table 1. This permits clustering and detection of SNPs in even highly expressed genes. The pipeline allows for smooth data transition between the different components/steps through the implementation of data interfaces that translate the output data format to format required in the next step, parse outputs, and allow the user to identify and select subsets of the output for the next step of the pipeline. Some of the filters that help verify a single nucleotide polymorphism as a candidate SNP have also been implemented here, so that the pipeline identifies high quality candidate SNPs.

In silico analysis revealed 184 interspecific SNPs in 15 contigs of *Cicer* ESTs derived from 26 different species. The program SNP2CAPS involves the screening of multiply aligned sequences for restriction sites followed by a selection pipeline that allows the deduction of CAPs candidates by the identification of putative alternative restriction patterns. Any primer pair flanking the SNP site then becomes suitable for CAPS marker analysis. In order to utilize the identified SNPs in chickpea genetics and breeding applications, 14 putative (and commonly used) restriction enzymes for assaying the SNPs were tested on 12 *Cicer* species using 17 primer-enzyme combinations. An unequivocal restriction pattern was observed in 5 (30%) primer enzyme combinations. The reason for not getting restriction patterns in 12 primer-enzyme combinations (70%) is attributed to not having been able to genotype all the species from which the SNPs were derived. In this study, we have tried to include all the *Cicer* species from the ICRISAT Genebank. Nevertheless, we anticipate that access to all species from which the public EST dataset was derived, will allow the entire restriction pattern to be visualized. For further validation, analysis of sequences from 11 genotypes derived with one primer pair clearly allows visualization of the restriction site for the enzyme *Hae*III. Indeed, these two genotypes showed restriction when the amplicons generated in these genotypes were digested with the enzyme. A study of the sequences of the three ESTs (AF522079, AF522081, and AY386897) from which this SNP was derived shows the existence of another restriction site for *Hae*III in the region; however, the sequence data available for the 11 amplicons does not cover this region. In summary, this study clearly confirms the identification and genotyping of true SNPs in the experiments.

The pipeline described in this manuscript differs from publicly available pipelines/workflows and workflow management systems (WMSs) like the BioWMS [18], KDE Biosciences [19], Taverna [20], Biopipe [21], GMP platform [22], and Biowep [23]. All of these are data integration and analysis frameworks that allow a well-informed and skilled bioinformaticist to create workflows using resources (both data and analytical tools) that are available as web services and also to provide web service access to their own database and tools to share with the community. The SNP discovery pipeline could constitute a workflow in any of the above platforms. There are similarities with this pipeline and the Biopipe and GMP platform in that they are meant to run high throughput bioinformatics analysis in a distributed computing environment. Both these tools require considerable programming skills on the part of the user. The approach of our pipeline has been quite simple, and providing access through user interfaces allowing anyone skilled in using interfaces to define what he would like to do within the pipeline, execute and manage results; without knowledge of the cluster environment he is interfacing with. Analysis components include parsers and wrappers like the Biopipe tool. Our application can be part of a workflow in a distributed computing repository. We anticipate that the pipeline to mine and assay SNPs using the cost-effective CAPS platform would be of considerable interest to the plant genetics and breeding community. The pipeline is available to academic users upon request.

## Figures and Tables

**Table 1:** Test datasets for the pipeline, output, and time taken (running on four 64-bit dual AMD opteron nodes of the Paracel high-performance linux cluster).

Species	Size of EST dataset	Number of clusters^a^	Maximum size of cluster	Minimum size of cluster	Average size of cluster^b^	Number of contigs^c^	Total SNPs identified^d^	Indels^d^	Total time taken^e^
Wheat	306699318 bp (579879 seq.)	27461	268269	2	9.769	39280	12217	10734	11 h 16 min
Maize	183675067 bp (407423 seq.)	22650	125014	2	5.5193	28008	10780	7822	7 h 35 min
Soybean	135866187 bp (330436 seq.)	22043	99619	2	4.5193	34622	7423	8178	7 h 4 m
*Sorghum*	124381970 bp (227587 seq.)	18488	54471	2	2.9462	21599	9463	8700	4 h 30 min
*Medicago*	121146352 bp (226923 seq.)	16151	36053	2	2.2322	23839	6942	16319	4 h 2 min
*Phaseolus*	2596245 bp (48334 seq.)	5537	1877	2	0.3389	4558	2086	1110	1 h
*Arachis*	8357124 bp (14381 seq.)	1473	2517	2	1.7087	1180	454	532	22 min
Rye	4342748 bp (9253 seq.)	1295	174	2	0.1343	568	218	86	18 min
Millet	1486253 bp (3106 seq.)	440	184	2	0.4181	135	28	35	3 min
Pigeonpea	428564 bp (925 seq.)	88	14	2	0.1590	6	4	1	1 min

^a^Output of the first step of the pipeline, namely, clustering with parallelized MegaBlast.

^b^Average size of cluster = maximum size of cluster/number of clusters.

^c^Number of contigs derived from PCAP output.

^d^SNPs and indels identified from the polybayes output file by custom scripts.

^e^Total time taken from EST file upload to SNP2CAPS output.
